# Prevalence of *Rickettsia* spp. Infection in Ticks Collected from Shelter Dogs in Tulcea County, Romania

**DOI:** 10.3390/pathogens15010036

**Published:** 2025-12-26

**Authors:** Bianca-Lavinia Andronic, Larisa-Maria Ivănescu, Gabriela-Victoria Martinescu, Raluca Mîndru, Liviu Miron

**Affiliations:** Department Clinics, Faculty of Veterinary Medicine, “Ion Ionescu de la Brad” Iasi University of Life Sciences, 8 Mihail Sadoveanu Alley, 700490 Iaşi, Romania; gabriela.martinescu@iuls.ro (G.-V.M.); raluca.mindru@iuls.ro (R.M.); liviu.miron@iuls.ro (L.M.)

**Keywords:** *Rickettsia* spp., *Ixodid* ticks, positivity rate, shelter dogs, molecular detection

## Abstract

Ticks are one of the most important vectors of zoonotic pathogens in Europe, including *Rickettsia* spp. In Romania, several pathogenic species belonging to the Spotted Fever Group (SFG) transmitted primarily through tick bites have been identified. The aim of the present study was to determine the prevalence of *Rickettsia* spp. infection in ticks collected from public shelter dogs in Tulcea County, Romania, as an indicator of pathogen circulation within shelter environments and their potential epidemiological relevance. A total of 730 ticks collected between March 2023 and September 2024 from 216 public shelter dogs, belonging to four species (*Rhipicephalus sanguineus* s.l., *Ixodes ricinus*, *Dermacentor marginatus*, and *Dermacentor reticulatus*), were morphologically identified and pooled by species, sex, and developmental stage resulting in 132 pools. DNA extracted from pools was tested by Real-Time PCR, targeting the *gltA* gene, specific for *Rickettsia* spp. Overall, 123/132 (93.2%) pools tested positive for *Rickettsia* spp. DNA, while the Minimum Infection Rate (MIR) at tick level was 16.8%. High pool positivity rates were consistently observed across all four species: 93.6% positive pools for *Rhipicephalus sanguineus* s.l. (MIR 15%), 88.2% for *Ixodes ricinus* (MIR 20.5%), 87.5% for *Dermacentor marginatus* (MIR 58.3%), and 100% for *Dermacentor reticulatus* (MIR 21.7%). These findings demonstrate a substantial circulation of *Rickettsia* spp. in dog-shelter associated ticks, highlighting the role of dogs as sentinel hosts. Continuous molecular surveillance and identifying circulating species is needed for a better determination of the zoonotic risk.

## 1. Introduction

Ticks (Acari: Ixodidae) are hematophagous ectoparasites that feed on a broad spectrum of hosts [1] and act as vectors of multiple pathogens (e.g., bacteria, viruses, helminths and protozoa) [2,3]. In Europe, ticks are considered the primary vectors of human and animal diseases, followed by mosquitoes. Tick-borne diseases (TBDs) represent an increasing public health and veterinary concern, with expanding geographical distribution driven by environmental change and intensified human–animal interactions [4,5]. Climate change, urbanization, changes in land-use patterns and increased mobility of humans and animals have contributed to the rising incidence of TBDs and have intensified scientific interest in this field. These factors have also facilitated the emergence of new tick species in non-endemic areas, along with the appearance of previously unreported TBDs [6].

In Romania, 25 species of hard ticks were identified, belonging to the genera *Ixodes*, *Haemaphysalis*, *Rhipicephalus*, *Dermacentor*, and *Hyalomma* [7,8]. *Ixodes ricinus* is the dominant species in Romania, with a prevalence of 86.9% [9], and it is also the most widespread species in Europe [10,11]. This species is a known vector of causative agents of numerous diseases such as babesiosis, Lyme borreliosis, tick-borne encephalitis (TBE), rickettsiosis, and anaplasmosis [12]. In dogs, TBDs represent a significant concern, because of the diagnostic challenges, the variable clinical manifestations, and the potential for co-infections [1]. Most of the TBDs transmitted to dogs are zoonotic, which increases the risk of transmission to humans. In urban settings, dogs contribute to the persistence of tick populations and infected ticks, thereby indirectly supporting the circulation of tick-borne pathogens due to their frequent exposure to infestations [2,13].

Bacteria of the genus *Rickettsia* are Gram-negative, obligate intracellular bacteria that reside within the cytosol of the host cell. These bacteria are encountered worldwide and are transmitted through arthropod vectors such as ticks, fleas, lice and mites [14,15]. The genus *Rickettsia* is taxonomically divided into four groups: the ancestral group (with non-pathogenic species), the Spotted Fever Group (SFG), the typhus group, and the transitional group. The latter three groups include species capable of causing diseases. The SFG and the typhus group represent the classical clades, while the transitional group shares characteristics of both [15,16,17]. Advances in molecular techniques have led to the identification of multiple new species, mainly in the SFG, of which at least fifteen species are known to be pathogenic [18,19,20]. The SFG rickettsiae are transmitted during tick feeding, while typhus group rickettsiae are transmitted via the contact of a skin wound or mucous membrane with infected feces of flea or lice [4,21]. Once inoculated, the pathogens are phagocytosed by dendritic cells, transported to regional lymph nodes to replicate, and subsequently disseminate in the blood stream to infect vascular endothelial cells. The endothelial damage leads to increased vascular permeability and clinical manifestations that range from mild to severe, including rash, interstitial pneumonia, meningoencephalitis, multiple organ dysfunction, or death [15,21,22,23].

Several pathogenic *Rickettsia* species have been identified in Romania, all belonging to the Spotted Fever Group (SFG), including *Rickettsia helvetica*, *Rickettsia monacensis*, *Rickettsia hoogstraalii*, *Rickettsia massiliae*, *Rickettsia raoultii,* and *Rickettsia slovaca* [2,4,9,12,24,25,26]. These species are transmitted through the bite of hard ticks belonging to the genera *Ixodes*, *Rhipicephalus*, *Dermacentor*, and *Haemaphysalis* [21]. In addition, *Rickettsia conorii* subsp. *conorii*—the causative agent of Mediterranean spotted fever (MSF)—has been a notifiable disease in Romania since 2000 [24], with the highest endemicity reported in Constanța, Tulcea, and Bucharest. This species is one of the most clinically important SFG rickettsiae and is primarily transmitted by *Rhipicephalus sanguineus* (the brown dog tick) [26,27].

In this context, dogs represent a particularly important host. As companion animals, they frequently live in close contact with humans, acting as sentinel hosts for rickettsioses [7,13]. Furthermore, in public shelters, dogs are often housed in crowded conditions, where heavy tick infestations are common and routine external deparasitation may be inconsistent [19]. Such an environment creates an opportunity for pathogen maintenance and amplification, increasing the transmission risk for the human population through tick bites [9,19].

Although several studies have reported the presence of *Rickettsia* spp. in questing ticks from vegetation, data regarding ticks directly collected from dogs in public shelters remain scarce. This is especially relevant for Tulcea County, a region with ecological characteristics favorable for tick development, high densities of stray dogs and documented endemicity for rickettsiosis.

This study aimed to investigate the prevalence of *Rickettsia* spp. in ticks collected from public shelter dogs in Tulcea County (Romania), using pool positivity rates and minimum infection rates (MIR) as indicators of pathogen circulation within shelter environments. By screening ticks from different species using Real-Time PCR, the study provides relevant information of the presence of *Rickettsia* spp. in a setting characterized by high infestation pressure and frequent animal–human contact.

## 2. Materials and Methods

### 2.1. Tick Collection, Morphological Identification and Pooling Procedure

Between March 2023 and September 2024, a total of 730 ticks were collected directly from 216 dogs housed in one public shelter of Tulcea County, Romania. Of the examined dogs, 137 were females and 79 were males; all were mixed-breed, with ages ranging from 6 months to 18 years, and originated from Tulcea County. Routine group-level ectoparasite treatments are applied at the shelter. Sampling was performed once for each dog, during routine veterinary inspections carried out at admission or during spay and neuter campaigns, immediately after restraining the animals to prevent detachment and potential loss of ticks. No ethical approval was required, as ticks were collected during routine veterinary procedures without additional manipulation of animals. Ticks were manually removed with fine-tipped forceps, placed into sterile 1.5 mL Eppendorf tubes labeled with the collection date and dog identification number. The tubes were stored initially at 4 °C for transportation to the laboratory and subsequently preserved in 70% ethanol until further processing.

All specimens were morphologically identified using standard taxonomic keys and dichotomous identification criteria [10,28,29] under a Zeiss Stemi 305 stereomicroscope (Zeiss, Oberkochen, Germany). After identification, ticks were pooled by species, sex, and developmental stage (adult males, adult females, and nymphs), with up to 17 ticks per pool to increase the amount of genetic material and optimize processing time. The average number of ticks per pool was 5.53 (730 ticks divided into 132 pools). Due to the engorgement status of many female ticks, female pools contained fewer specimens, whereas male and nymph pools generally included a higher number of specimens (Table 1). Morphological identification and molecular analyses were performed at the Molecular Biology Laboratory of the Department of Parasitic Diseases, Faculty of Veterinary Medicine, Iași.

### 2.2. DNA Extraction

Molecular screening began with DNA extractions from pools. DNA extractions were performed using the AllPrep DNA/RNA Mini Kit (Qiagen, Hilden, Germany), according to the manufacturer’s protocol. Ticks were surface-sterilized in 70% ethanol before DNA extraction to remove potential external contaminants [30]. For each pool, approximately 200 µL of digested material was processed. Samples were first lysed and homogenized in RLT Plus buffer. The lysates were vortexed and centrifuged briefly to remove debris, then transferred to AllPrep DNA spin columns. After the selective binding of double-stranded genomic DNA, the columns were washed twice with washing buffers to remove proteins, salts, and other contaminants. DNA was subsequently eluted in 50 µL of nuclease-free water. Sample extractions were performed under sterile conditions, using a laminar flow hood to avoid contamination and also using negative controls. DNA concentration and purity were determined by measuring the absorbance at 260/280 nm using a NanoDrop One Spectrophotometer (ThermoFisher Scientific, Waltham, MA, USA). Template DNA concentrations ranged approximately from 100 to 1100 ng/µL, depending on pool size, sex composition, and feeding status, providing sufficient yield for downstream qPCR amplification. Extracted DNA was stored at −20 °C until further use in molecular assays.

### 2.3. Molecular Detection: Real-Time Polymerase Chain-Reaction (qPCR)

A Real-Time PCR assay targeting a conserved region of the citrate synthase gene (*gltA*) of *Rickettsia* spp. [31] was used for molecular detection. The qPCR protocol, as previously described by Stenos et al. (2005) [32], included the primer-probe set: CS-F (5′-TCG CAA ATG TTC ACG GTA CTT T-3′), and CS-R (5′-TCG TGC ATT TCT TTC CAT TGT G-3′) and the probe CS-P (5′-6-FAM-TGC AAT AGC AAG AAC CGT AGG CTG GAT G-BHQ-1-3′). Each PCR reaction was carried out in a total volume of 25 µL, containing 200 nM of each primer and probe, 2× Platinum™ Quantitative PCR SuperMix-UDG (ThermoFisher Scientific, USA), 5 mM MgCl_2_, 2–5 µL of template DNA and nuclease-free water to volume [32].

Amplification was performed on Bio-Rad CFX96™ Real-Time Detection System (Bio-Rad, Hercules, CA, USA). The thermal conditions consisted of 95 °C for 5 min, followed by 60 cycles of 95 °C for 20 s and 60 °C for 40 s. Each run included positive and negative controls, and results were analyzed using CFX Manager™ Software Version 3.1 (Bio-Rad, Hercules, CA, USA). Samples with a cycle threshold (Ct) value < 35 were considered positive.

### 2.4. Data Analysis

The prevalence of *Rickettsia* spp. infection in ticks was estimated using two complementary measures. The Minimum Infection Rate (MIR) was calculated as:

MIR=p/N×100%

where *p* is the number of positive pools and *N* is the total number of ticks tested. This approach expresses the minimum proportion of infected specimens in pooled samples and assumes that only one infected tick is present in each positive pool [33].

In addition, the Pooled Positivity Rate (PPR) [34] was calculated as the proportion of positive pools out of the total number of pools tested for each tick species, sex, and developmental stage. This measure reflects the frequency of *Rickettsia* DNA at the pool level.

## 3. Results

A total of 730 ticks were collected from 216 public shelter dogs (137 females and 79 males, all mixed breeds, aged between 6 months and 18 years) in Tulcea County during the study period. Tick burden per dog ranged from 1 to 17 ticks, with a mean of 3.38 ticks per dog (median = 2; SD = 3.02). Four tick species were identified: *Rhipicephalus sanguineus* s.l. (*n* = 585), *Ixodes ricinus* (*n* = 73), *Dermacentor marginatus* (*n* = 12), and *Dermacentor reticulatus* (*n* = 60). After pooling by species, sex and developmental stage, a total of 132 pools were created: 94 pools of *R. sanguineus* s.l. (58 female, 32 male and 4 nymph pools), 17 pools of *I. ricinus* (15 female and 2 male pools), 8 pools of *D. marginatus* (6 female and 2 male pools), and 13 pools of *D. reticulatus* (9 female and 4 male pools).

**Figure 1:**
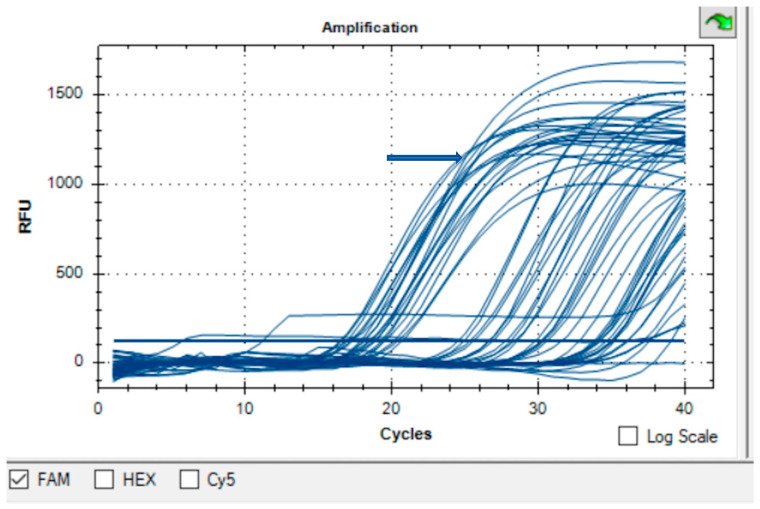
Amplification curves obtained by Real-Time PCR for the detection of *Rickettsia* spp. DNA in 132 tick pools. The blue arrow highlights the exponential amplification curves detected on the FAM channel, corresponding to the positive pools (RFU—relative fluorescence units).

Molecular screening by Real-Time PCR targeting the *gltA* gene detected *Rickettsia* spp. in 123/132 pools, corresponding to an overall pool positivity of 93.2% (Figure 1). Species-level positivity rates are shown in Table 2 and are summarized as follows:

- *Rhipicephalus sanguineus* s.l.: 88/94 pools (93.6%)
- *Ixodes ricinus*: 15/17 pools (88.2%)
- *Dermacentor marginatus:* 7/8 pools (87.5%)
- *Dermacentor reticulatus*: 13/13 pools (100%)

In addition to the Pool Positivity Rate (PPR), the Minimum Infection Rate (MIR) was calculated to estimate the proportion of infected ticks within each species, under the assumption that each positive pool contains at least one infected specimen. The MIR values were 15% for *R. sanguineus* s.l., 20.5% for *I. ricinus*, 58.3% for *D.marginatus*, and 21.7% for *D.reticulatus*. Overall, the MIR across all ticks was estimated at 16.8% (Table 3).

These findings indicate a high prevalence of *Rickettsia* spp. across all four tick species collected from shelter dogs in the study area.

## 4. Discussion

The results obtained in this study highlight a very high positivity rate for *Rickettsia* spp. in ticks collected from dogs housed in the public shelter of Tulcea County, with an overall pool positivity of 93.2% and a minimum infection rate (MIR) of 16.8%. These values are comparable to, or even higher than, those previously reported in eastern Romania, where prevalence ranged between 10% and 23.8% [4,9]. The higher pool positivity rates observed here may be related to the fact that all ticks were collected directly from dogs with intense and repeated exposure to infestation, rather than from vegetation, as in most previous surveys.

An important finding of this study is the consistently high detection of *Rickettsia* spp. across all four tick species identified (*R. sanguineus* s.l., *I. ricinus*, *D. marginatus,* and *D. reticulatus*). Similar detection of *Rickettsia* spp. in multiple tick species has been reported in previous studies from Romania and other European countries, highlighting the broad circulation of spotted fever group rickettsiae in domestic and peri-domestic settings [2,4,9,19]. This pattern suggests that multiple tick species associated with shelter dogs may contribute to the maintenance and circulation of *Rickettsia* spp. in this setting. While *R. sanguineus* s.l. and *I. ricinus* are known vectors for several spotted fever group rickettsiae in Europe [21,23,24,27], the present results cannot confirm their involvement in the transmission of a specific *Rickettsia* species, as only genus-level detection was performed. The high MIR values recorded for *D. marginatus* and *D. reticulatus* further indicate substantial exposure of shelter dogs to ticks harboring *Rickettsia* spp. Comparable positivity levels in *Dermacentor* ticks have been reported in Romania and other parts of Central and Eastern Europe, where *R. raoultii* and *R. slovaca* are frequently detected [4,21]. However, lower prevalence values have been observed in studies based on questing ticks collected from vegetation [9,19], suggesting that host-associated sampling, high infestation pressure, and local ecological conditions may significantly influence observed pool positivity rates.

The increased prevalence of infection in the shelter environment—characterized by high dog density, massive infestation pressure and irregular ectoparasite control—suggests that such settings may act as foci for the persistence and amplification of tick-borne pathogens [35]. In this context, dogs can be considered sentinel hosts, reflecting pathogen circulation in environments with frequent human–animal contact [36].

However, ticks may acquire rickettsial DNA through co-feeding transmission, a mechanism in which pathogens pass locally between feeding ticks without requiring systemic infection of the vertebrate host [37]. In such situations, a tick may test positive for *Rickettsia* spp. without being a competent vector for that particular species. Therefore, the findings of this study should be interpreted as evidence of exposure to *Rickettsia* spp. within the shelter environment. The use of MIR assumes that each positive pool contains only one infected tick, likely underestimating the true prevalence.

Despite these limitations, the results support the need for continuous molecular surveillance of ticks and shelter dogs, as well as further investigations aimed at species-level identification of circulating *Rickettsia*. Implementing rigorous ectoparasite control programs in public shelters could help reduce the associated zoonotic risk.

## 5. Conclusions

This study confirms the presence and circulation of *Rickettsia* spp. in ticks collected from dogs in a public shelter from Tulcea County, highlighting the potential epidemiological role of shelter dogs as sentinels for pathogen monitoring in environments with high tick infestation pressure.

Species-level identification of *Rickettsia* and complementary investigations are needed to clarify which rickettsiae are circulating and to better understand their potential veterinary and public health significance. Integrating such data into One Health surveillance programs is essential to more accurately assess zoonotic risks and to support effective prevention and control measures.

## Figures and Tables

**Table 1:** Number of ticks and pool composition by species, sex, and developmental stage.

Tick Species	No. of Ticks	Females	Female Pools	Males	Male Pools	Nymphs	Nymph Pools
*R. sanguineus* s.l.	585	232	58	324	32	29	4
*I. ricinus*	73	63	15	10	2	0	0
*D. marginatus*	12	8	6	4	2	0	0
*D. reticulatus*	60	30	9	30	4	0	0
Total	730	333	88	368	40	29	4

**Table 2:** Summary of tick pools tested and pool positivity rates (PPR) for *Rickettsia* spp. DNA.

Tick Species	Pools Tested (*n*)	Positive Pools (*n*)	PPR (%)	Females (Pos/Total)	PPR (%)	Males (Pos/Total)	PPR(%)	Nymphs (Pos/Total)	PPR (%)
*R. sanguineus* s.l.	94	88	93.6%	54/58	93.1%	30/32	93.8%	4/4	100%
*I. ricinus*	17	15	88.2%	14/15	93.3%	1/2	50%	-	
*D. marginatus*	8	7	87.5%	5/6	83.3%	2/2	100%	-	
*D. reticulatus*	13	13	100%	9/9	100%	4/4	100%	-	
Total	132	123	93.2%	82/88	93.2%	37/40	92.5%	4/4	100%

**Table 3:** Comparison of Pool Positivity Rate (PPR) and Minimum Infection Rate (MIR) of *Rickettsia* spp. in ticks collected from dogs.

Tick Species	No. of Ticks	Pools Tested	Positive Pools	PPR (%)	PPR 95% CI	MIR (%)	MIR 95% CI
*R. sanguineus* s.l.	585	94	88	93.6	86.6–97.6	15.0	12.2–18.2
*I. ricinus*	73	17	15	88.2	63.6–98.5	20.5	12.0–31.6
*D. marginatus*	12	8	7	87.5	47.3–99.7	58.3	27.7–84.8
*D. reticulatus*	60	13	13	100	75.3–100	21.7	12.1–34.2
Total	730	132	123	93.2	-	16.8	-

## Data Availability

The data of this report are available from the corresponding authors upon request.

## Abbreviations

The following abbreviations are used in this manuscript:

DNA	Deoxyribonucleic acid
MIR	Minimum Infection Rate
PCR	Polymerase Chain Reaction
PPR	Pool Positivity Rate
RNA	Ribonucleic acid
SFG	Spotted Fever Group
TBDs	Tick-borne Diseases
